# DNA capture and next-generation sequencing can recover whole mitochondrial genomes from highly degraded samples for human identification

**DOI:** 10.1186/2041-2223-4-26

**Published:** 2013-12-02

**Authors:** Jennifer E L Templeton, Paul M Brotherton, Bastien Llamas, Julien Soubrier, Wolfgang Haak, Alan Cooper, Jeremy J Austin

**Affiliations:** 1 Australian Centre for Ancient DNA, School of Earth and Environmental Sciences, The University of Adelaide, Adelaide, South Australia 5005, Australia; 2 School of Biological Sciences, Flinders University, Bedford Park, Adelaide, South Australia 5001, Australia; 3 Archaeogenetics Research Group, School of Applied Sciences, University of Huddersfield, Queensgate, Huddersfield HD1 3DH, UK; 4 Sciences Department, Museum Victoria, Carlton Gardens, Melbourne, Vic 3001, Australia

**Keywords:** Mitochondrial DNA, Degraded DNA, Ancient DNA, DNA hybridisation, DNA enrichment, Forensic science, Next-generation sequencing

## Abstract

**Background:**

Mitochondrial DNA (mtDNA) typing can be a useful aid for identifying people from compromised samples when nuclear DNA is too damaged, degraded or below detection thresholds for routine short tandem repeat (STR)-based analysis. Standard mtDNA typing, focused on PCR amplicon sequencing of the control region (HVS I and HVS II), is limited by the resolving power of this short sequence, which misses up to 70% of the variation present in the mtDNA genome.

**Methods:**

We used in-solution hybridisation-based DNA capture (using DNA capture probes prepared from modern human mtDNA) to recover mtDNA from post-mortem human remains in which the majority of DNA is both highly fragmented (<100 base pairs in length) and chemically damaged. The method ‘immortalises’ the finite quantities of DNA in valuable extracts as DNA libraries, which is followed by the targeted enrichment of endogenous mtDNA sequences and characterisation by next-generation sequencing (NGS).

**Results:**

We sequenced whole mitochondrial genomes for human identification from samples where standard nuclear STR typing produced only partial profiles or demonstrably failed and/or where standard mtDNA hypervariable region sequences lacked resolving power. Multiple rounds of enrichment can substantially improve coverage and sequencing depth of mtDNA genomes from highly degraded samples. The application of this method has led to the reliable mitochondrial sequencing of human skeletal remains from unidentified World War Two (WWII) casualties approximately 70 years old and from archaeological remains (up to 2,500 years old).

**Conclusions:**

This approach has potential applications in forensic science, historical human identification cases, archived medical samples, kinship analysis and population studies. In particular the methodology can be applied to any case, involving human or non-human species, where whole mitochondrial genome sequences are required to provide the highest level of maternal lineage discrimination. Multiple rounds of in-solution hybridisation-based DNA capture can retrieve whole mitochondrial genome sequences from even the most challenging samples.

## Background

Nuclear DNA short tandem repeat (STR) profiling is currently the preferred method for human identification in forensic practice [1]. However, analysis of low copy number (LCN) and highly damaged or degraded DNA from trace sources or poorly preserved human remains is challenging due to stochastic effects and can often fail completely [2]. Typical complications observed in the analysis of trace amounts of DNA include issues with contamination, amplification failure (allele and locus dropout) [3], preferential amplification of shorter amplicons [4] and artefacts (enzymatic stutter, allele drop-in and off-ladder peaks). Complete amplification failure can be due to PCR inhibition or the fragmentation of all DNA templates below target amplicon sizes, which generally range from 100 to 400 base pairs (bp) [5]. Another complication – ‘jumping PCR’ – can generate non-authentic chimeric amplicons from discrete DNA template molecules, particularly when DNA fragmentation levels are high [6,7]. Additionally, chemical DNA modification due to miscoding lesions can terminate amplification reactions by halting DNA polymerase extension [7]. A combination of all these factors can lead to a poor or misleading DNA profile, or no profile at all in extreme cases.

The development and optimisation of nuclear SNP (single nucleotide polymorphism) typing protocols, shorter amplicon commercial STR kits (mini-STRs) [8], optimisation of PCR conditions, capillary electrophoresis and statistical interpretation techniques [9] have improved standard profiling methods [10-12]. However, in spite of these developments, the STR profiling of degraded, low-template DNA often has limited success. Furthermore, a large number of nuclear SNPs are required (50 to 80 loci) to obtain a similar level of discrimination as a full nuclear 16-loci STR profile [13]. In these cases, genetic identification from degraded samples may succeed through the analysis of mitochondrial DNA (mtDNA).

Mitochondrial DNA has several features that can make it a useful marker for human identification. As there can be thousands of copies of the mitochondrial genome in many cells (compared to only two copies for autosomal nuclear DNA), mtDNA typing is well suited to biological specimens where DNA fragmentation has occurred or the total DNA copy numbers are naturally low or have been severely reduced through post-mortem damage and degradation [14]. Suitable materials include bones, teeth, hair shafts, faeces and other biological materials. The lack of recombination events in the mtDNA genome and strict uniparental inheritance, in contrast to the nuclear genome, can allow maternal relatives separated by several generations to serve as reference samples. This latter feature is particularly beneficial in missing-person identification, where suitable ante-mortem or family reference samples may be unavailable.

Standard PCR-based sequencing approaches for mitochondrial hypervariable regions I and II (HVS I and II) typically amplify 2 to 12 overlapping fragments of approximately 150 bp to 600 bp in length [15-18] but are labour intensive, consume significant amounts of valuable DNA extract and can be template-length dependent and costly. Repeated singleplex PCR amplifications also bring an increased risk of contamination with exogenous human DNA due to the multiple lab steps required. Multiplex PCR amplification could in theory provide a solution for medium-sized PCR target fragments but still require hundreds of overlapping amplicons [1] in cases where whole mitochondrial genome sequences are needed for high-resolution identification.

Another disadvantage of typing just the mitochondrial HVS I/II is that short sequences from this single locus are far less powerful for identification purposes than a full multi-locus STR profile [19]. This can become a significant problem when many individuals in a population share a common haplogroup, such as the >40% of Western Europeans who belong to mitochondrial haplogroup H, or when distantly related individuals share a maternal ancestry that may not be known [20]. Recent studies sequencing whole mitochondrial genomes have shown that >70% of the mtDNA variation can be located outside HVS I/II for some haplogroups [21], so that full mitochondrial genome sequences provide far greater resolving power for human identification [22,23].

Ancient DNA studies of human archaeological samples routinely generate complete mitochondrial genomes via DNA hybridisation-based enrichment of mtDNA target sequences [21,24-26], and the creation of barcoded/indexed DNA libraries, followed by next-generation sequencing (NGS). Multiple samples can be processed in parallel in a high-throughput fashion [25], greatly reducing processing contamination risks, labour and costs compared to traditional Sanger sequencing approaches. These kinds of DNA capture strategies generally rely on the hybridisation of target DNA sequences to probes that are either immobilised on a surface (such as a microarray) or in solution [27,28]. Despite the significant potential of these new approaches, they have not been applied or examined in a forensic context for human identification.

**Figure 1:**
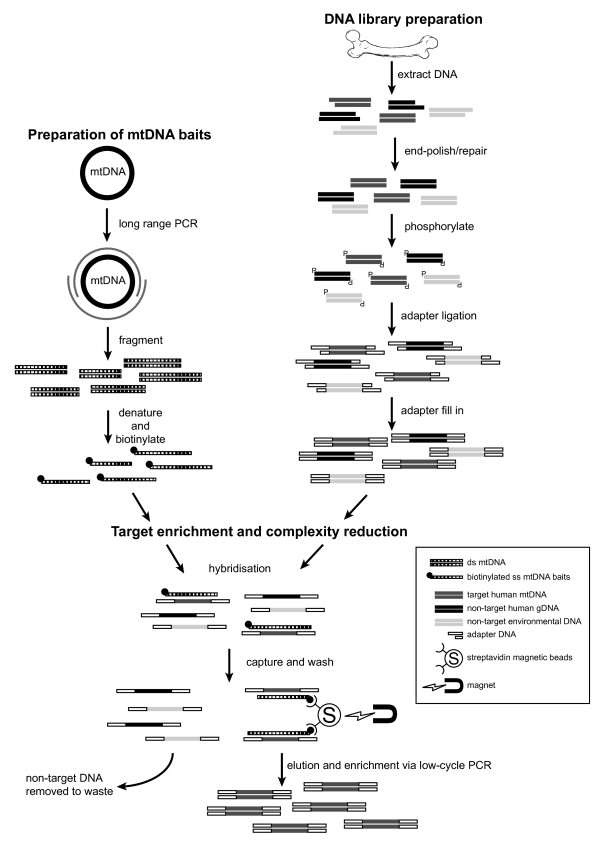
**mtDNA hybridisation enrichment protocol.** mtDNA baits were prepared using 2 × 8 kb mtDNA long-range PCR products (spanning 16,569 base pairs), generated using a DNA extract from a present-day sample (of a known haplotype) and the Roche LR Expand PCR kit. The products were fragmented by physical shearing to create 200 bp to 600 bp fragments prior to end labelling with biotin. The DNA library was prepared as follows. Damaged DNA leaves 5′ and 3′ overhangs. T4 DNA polymerase was used to polish the DNA by creating blunt ends and T4 PNK phosphorylated 5′ ends, which is required for adaptor ligation. T4 ligase attached universal hybridisation adaptors (Uni-hyb A and Uni-hyb B) to the phosphorylated ends. Klenow polymerase filled in the short-arm adaptor ligation to create double-stranded adaptors (through the use of deoxyribonucleotide triphosphates - dNTPs). Adaptor complementary primers and Taq polymerase amplified the entire library to immortalise the sample. Single-stranded probe DNA was mixed with single-stranded library DNA and left to hybridise overnight (in the presence of blocking oligos). Biotinylated probe and bound library DNA were fixed to streptavidin beads on a magnetic rack, and non-specific or weakly bound library DNA was washed away through a series of three stringency washes (by increasing temperature and decreasing salt concentration) from the library–probe–streptavidin interaction. The single-stranded library DNA was converted to double stranded DNA and eluted from the probe–streptavidin interaction using the Bst strand-displacing enzyme (in the presence of dNTPs). Bst recognises nicks in the template and displaces library DNA into solution. Probe DNA remained bound to the magnet. Eluted library DNA was enriched through low cycle PCR, using adaptor complementary primers. Library DNA was then prepared for next-generation sequencing. mtDNA, mitochondrial DNA; PCR, polymerase chain reaction.

**Figure 2:**
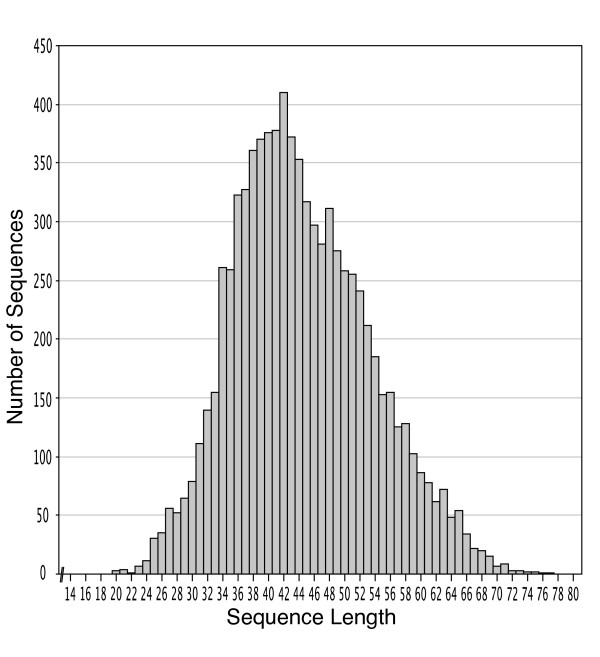
**Distribution of the length of human mtDNA reads from sample ID 8727C.** In total, 277,470 reads were trimmed to remove primers and the resulting 218,102 mapped (to the Reconstructed Sapiens Reference Sequence) reads were filtered to remove duplicates as a result of clonal reads. The resulting 7,756 unique mapped reads (2.8% of the total reads) gave 99.5% mitochondrial genome sequence (16,487 base pairs) coverage. The mean fold coverage was 20x (SD ± 9.7), mean unique read length was 43.2 bp (SD ± 9.3) and all fragments captured were <80 bp.

The aim of this study was to sequence whole mitochondrial genomes from a range of human skeletal samples (in this case ranging from 10 to 2,500 years old) at an affordable cost using standard laboratory equipment and home-made DNA-capture probes for use in hybridisation-based target enrichment (Figure 1). Our previous application of this method [21] used three rounds of in-solution capture-based enrichment so we also aimed to explore the efficacy of using one or two rounds of enrichment to reduce costs and improve workflows. We deliberately used samples that had previously failed or had the potential to fail nuclear STR typing (Table 1). STR profiling was predominantly performed to assess the likelihood of obtaining full STR profiles from degraded samples and not to identify the samples. To identify the samples would require reference profiles for comparison and replicate testing of the samples by LCN analyses. The capture-probe method is designed to focus on the recovery of human mtDNA fragments <100 bp in length (with the vast majority in the 20 to 70 bp range) (Figure 2), from samples that yield highly damaged and fragmented DNA templates available only with low copy number. We anticipate the method will be useful for samples that cannot be typed successfully using standard STR kits and for detecting key or private SNPs within whole mitochondrial genome sequences (that would otherwise remain undetected with traditional mtDNA HVS I/II sequencing) for human identification.

**Table 1:** Samples used in this study

**ACAD ID**	**Description**	**Age**	**Excavated**	**Locality**	**Environmental conditions of site**	**Preservation status on collection**
8727C	Cranium fragment	Approximately 10 years	Unknown	South-east Queensland, Australia	Surface deposit	Well preserved
11995A	Long bone fragment	Approximately 70 years	2011	Papua New Guinea	Tropical lowland battlefield, wet	Poor
9210A	Tooth	Approximately 70 years	2006	Christmas Island, Australia	Tropical lowland, burial, wet	Well preserved
4464B	Long bone fragment	Approximately 2500 years (Iron Age)	2007	Latsch, South Tyrol, Italy	Temperate, burial	Well preserved
10730A	Long bone fragment	Approximately 600 to 1,000 years (Ychsma culture)	2002	Huaca Pucllana, Lima, Peru	Temperate, high altitude, burial	Well preserved

ACAD, Australian Centre for Ancient DNA.

## Methods

### Samples

Bone and tooth samples were selected representing a range of ages, preservation conditions and contexts (Table 1). Three samples were from missing-person cases, two of which were from Australian servicemen killed in World War II. Two samples were recovered from archaeological contexts.

### Degraded DNA work

To avoid the potential for contamination of samples with contemporary human DNA or previously amplified PCR products, all steps preceding DNA library amplification were carried out in a dedicated ancient DNA laboratory geographically separated (by approximately 1.5 km) from post-PCR and other molecular biology laboratories at the University of Adelaide.

Strict decontamination procedures were followed [29]. There were ultraviolet lights in every room. There was positive air pressure and the one-way airflow was filtered using high efficiency particulate air filters. There were separate workrooms each containing dead-air glove boxes. Equipment and work areas were cleaned with sodium hypochlorite and isopropanol before and after use. Personnel protective clothing included a full-body suit, face mask, face shield, boots and triple-gloves. There was a strict one-way movement of personnel (from shower to freshly laundered clothes to ancient DNA laboratory to post-PCR laboratory).

Non-template controls and extraction blanks were included in each experiment to monitor potential contamination from exogenous human DNA sources and cross-contamination from other samples. The complete mitochondrial genomes of all staff involved in the handling of the samples (JT, WH, BL and PB) were sequenced to monitor potential contamination (Additional file 1: Table S1). The mitochondrial genome of an anonymised present-day sample used to generate mtDNA capture probes was also fully sequenced (haplotype: J1c8a) to monitor contamination (Additional file 1: Table S1).

### DNA extraction, quantification and STR profiling

DNA was extracted from five samples using an optimised method as previously described [21]; see Additional file 1.

For sample 9210A, a small quantity of DNA extract was available so only mitochondrial testing was performed. The four additional samples (4464B, 10730A, 8727C and 11995A) were subjected to both nuclear STR and mitochondrial sequence analyses. Nuclear DNA and mtDNA were quantified in all extracts using quantitative PCR (qPCR) with SYBR® green chemistry and previously published 67 bp nuclear [30] and 77 bp mitochondrial [31] PCR targets (Additional file 1: Table S5). The total 10 μL qPCR reaction mix consisted of 1× Brilliant II SYBR® green master mix (Agilent Technologies, USA), 0.15 μM forward primer (Additional file 1: Table S5), 0.15 μM reverse primer (Additional file 1: Table S5), 400 ng/μL rabbit serum albumin and 1 μL DNA extract. Samples were run in triplicate, and negative (no template) and positive controls (male genomic control DNA, Applied Biosystems, USA) were included in all runs. Extraction blank controls were also quantified. Cycling was performed using a Corbett 6000 Rotor-Gene real-time PCR thermocycler and consisted of an initial 5 min denaturation step at 95°C, followed by 45 cycles of 95°C for 10 s, 59°C for 20 s and 72°C for 15 s. Results were analysed using the Rotor-Gene 6000 Series Software 1.7. The DNA concentration was determined using the comparative cycle threshold method where unknown samples are compared to a standard curve. The standard curve for the nuclear target was created using male genomic control DNA (Applied Biosystems, USA). The standard curve for mitochondrial DNA was created using a PCR product (Additional file 1: Table S5).

STR typing was performed using AmpFLSTR ProfilerPlus™ (Applied Biosystems, USA). The final 12.5 μL reaction volume consisted of 4.6 μL ProfilerPlus™ reaction mix, 2.5 μL of ProfilerPlus™ primer mix, 0.4 μL AmpliTaq Gold™ and 5 μL of DNA extract. Cycling was performed on a 9700 GeneAmp thermal cycler and consisted of an initial denaturation at 95°C for 10 min followed by 34 cycles of 94°C for 1 min, 59°C for 1 min and 72°C for 1 min, then a final extension at 60°C for 45 min. PCR products were analysed on a 3130xl Genetic Analyser in a 17.3 μL final volume that consisted of 2 μL of PCR product, 15 μL HiDi™ formamide and 0.3 μL ROX-500 size standard (Applied Biosystems, USA). Results were analysed using Genemapper ID software (v3.2.1). Alleles were interpreted based on peak heights reaching a set threshold value of 25 relative fluorescence units (RFU) above a clean baseline. A wild-card designation was used, with peak heights <150 RFU, to account for potential allele dropout (for example, ‘11, F’ instead of ‘11,11’). A profile was considered full when all alleles were detected above the threshold RFU. A profile was defined as partial when peaks were detected above the threshold RFU and when at least one locus was successfully called. A profile in forensic terms can be described as partial when at least one locus has been called (even if this is not an informative profile).

### Mitochondrial DNA capture and enrichment

Biotinylated DNA-capture probes of a known haplotype and DNA libraries were generated as described in Additional file 1 and as previously described [21]. Whilst Brotherton *et al*. [21] used three rounds of enrichment for all samples, we explored the effects of using one or two rounds of enrichment on the number of unique reads, coverage and sequencing depth. Hybridisation was carried out in a final volume of 30 μL consisting of 100 ng of probe and 400 ng of library DNA. The thermal profile used was: denature DNA for 5 min at 95°C, followed by 14 to 18 hours incubation at 50°C to allow the DNA-capture probes to hybridise to fragments of DNA with closely matched sequences from complementary human DNA regions. The two library primers (Additional file 1: Table S4) were included as part of the hybridisation mix at 0.67 μM to 1.0 μM, to act as ‘blocking’ oligonucleotides. The blocking oligonucleotides are complementary to the library adaptors and have a dual role during the hybridisation reaction: (i) to minimise unwanted hybridisation between the adaptor-tagged flanking regions of otherwise unrelated single-stranded library DNA molecules and (ii) to enable strand displacement of probe DNA from library DNA as explained below.

Following overnight hybridisation at 50°C, 50 μL of magnetic streptavidin beads in solution (Invitrogen) were added to 30 μL of hybrid DNA and the beads were immobilised on a magnetic rack. The clear supernatant was discarded. The bead complex (DNA–capture probe/library DNA) immobilised to the magnet was subjected to successively increased-stringency washes, to remove progressively non- or weakly-hybridised single-stranded library DNA molecules, using decreased salt and increased temperature: 2× saline sodium citrate (SSC)/0.1% sodium dodecyl sulphate (SDS) at 37°C for 1 min; 2× SSC/0.1% SDS at 42°C for 10 min; 1× SSC/0.1% SDS at 43°C for 10 min; 0.5× SSC/0.1% SDS at 44°C for 10 min; 0.5× SSC/0.1% SDS at 45°C for 10 min.

The strand-displacing Bst DNA polymerase enzyme (large fragment, New England Biolabs) was used to release library DNA from the DNA-capture probe (immobilised to beads on the magnet). Reactions were performed at 35 μL final volume comprising 1× Thermopol buffer (New England Biolabs), 200 μM of each dNTP (to convert single-stranded library DNA to dsDNA), and 100 μg/mL of Bovine Serum Albumin (New England Biolabs). The reaction was pre-heated to 60°C and 2 μL of Bst enzyme was added last to each reaction. Tubes were incubated at 60°C for 5 min with regular agitation. The reaction tube was then applied to the magnetic rack at 60°C and 35 μL of supernatant was transferred to a fresh PCR tube. This tube was immediately incubated at 80°C for 20 min to inactivate the enzyme.

The heat-inactivated supernatant was split between eight PCR re-amplification reactions (total combined volume 140 μL), designed so that upon the addition of the sub-portion of Bst buffer, the final composition of the reactions would be 1× AmpliTaq Gold buffer II, 2.5 mM MgCl_2_, 250 μM of each dNTP, 1.0 U AmpliTaq Gold (Applied Biosystems), and 0.5 μM of PCR primers UniHyb-PCR-A and UniHyb-PCR-B (Additional file 1: Table S4). Thermocycling was at 94°C for 11 min, followed by 12 cycles of 30 s at 95°C, 30 s at 60°C and 1 min (+2 seconds per cycle) at 72°C, followed by a final 10 min at 72°C. Amplification reactions were pooled and library amplicons purified using MinElute spin columns (Qiagen) and eluted into 15 μL as per the manufacturer’s instructions. These comprised the ‘first enrichment’ DNA libraries and amplification products were sized and quantified via gel electrophoresis against size markers (HyperLadder™ V, Bioline) and a Nanodrop 2000 (Thermo Scientific).

For cases where a second round of enrichment took place, the overnight hybridisation and wash steps were repeated to produce ‘second enrichment’ DNA libraries highly enriched for mtDNA sequences.

### Ion Torrent PGM sequencing

Enriched library DNA was prepared for Ion Torrent sequencing by re-amplification using Ion Torrent barcoded primers (Additional file 1: Table S6). Eight 24 μL reaction volumes per sample were re-amplified using 1 μL of purified library DNA as the template. Final reactions conditions comprised of 1× AmpliTaq Gold buffer II, 2.5 mM MgCl_2_, 2.5 U AmpliTaq Gold (Applied Biosystems), 250 μM of each dNTP (Invitrogen), and 0.5 μM of each PCR primer. The thermocycling profile was 94°C for 12 min, followed by 12 cycles of 30 s at 95°C, 30 s at 60°C and 45 s at 72°C, followed by a final 10 min at 72°C. The eight amplified samples per reaction were pooled and purified using MinElute spin columns (Qiagen) and eluted into 15 μL as per the manufacturer’s instructions. The DNA was sized and quantified via gel electrophoresis against size markers (HyperLadder™ V, Bioline) and a Nanodrop 2000 (Thermo Scientific). Library DNA was size-selected above 120 bp and further purified to remove adaptor dimer, using Qiagen’s gel extraction purification kit following the manufacturer’s instructions.

Prior to sequencing, the fragment size distribution and DNA concentration of individual libraries were measured using a Bioanalyzer 2100 (Agilent Technologies) following the manufacturer’s instructions. The quantified indexed library DNA was pooled to an equimolar concentration prior to the One Touch. The pooled library DNA was adjusted to a final concentration of 10 to 15 pM prior to amplification (by emulsion PCR) and enriched for positive ion sphere particles (ISPs) using the Ion Torrent One Touch System II (Life Technologies) and the Ion One Touch 200 template kit v2 DL (Life Technologies), following the manufacturer’s instructions.

Templated ISPs were sequenced on a 316 micro-chip (up to 100 Mb of data expected) using the Ion Torrent Personal Genome Machine (PGM; Life Technologies) and the Ion PGM 200 sequencing kit v2 chemistry (Life Technologies) for 130 cycles (520 flows). After sequencing, the individual sequence reads were filtered within the PGM software to remove low-quality and polyclonal sequences. Sequences matching the PGM 3′ adaptor were also automatically trimmed prior to bioinformatics analysis.

### Bioinformatic sequence analysis

Ion Torrent PGM data from the mtDNA capture was processed using a customisable analytical pipeline. The scripts fastx_barcode_splitter.pl and fastx_trimmer (from the FASTX toolkit [32]) were used to demultiplex the reads by barcode, using a strict zero mismatch threshold. Cutadapt v1.1 [33] was then used to trim adapters using a maximum error rate of 0.33 (−e 0.3333), and to remove short (−m 25), long (−M 110) and low-quality sequences (−q 20), for a total of five passes (−n 5). The filtered reads were checked with FastQC [34] before being mapped against the Reconstructed Sapiens Reference Sequence (RSRS) [35] using TMAP v3.2.1 [36] with the following options: −g 3 -M 3 -n 7 -v stage1 --stage-keep-all map1 --seed-length 12 --seed-max-diff 4 stage2 map2 --z-best 5 map3 --max-seed-hits 10. The program TMAP has been optimised to align Ion Torrent PGM reads against a reference genome [37]. Mapped reads with mapping quality below Phred 30 and read duplicates were removed using Samtools v0.1.18 [38] and the MarkDuplicates tool of Picard Tools v1.79 [39]. The GC content of mapped reads was analysed using the CollectGcBiasMetrics tool of Picard Tools v1.79. Misincorporation patterns were assessed using mapDamage v0.3.6 [40]. The resulting sequence assembly was visualised using Biomatters Geneious Pro v5.6.2 software [41] and mitochondrial haplotypes were defined for each individual according to phylotree.org [4].

### Confirming SNP calls by hypervariable region I sequencing

HVS I was amplified using a minimum of four short overlapping primer pairs, as previously described [42,43]. Minisequencing of 22 coding region SNPs (GenoCoRe22) using a multiplex and SNaPshot based approach was carried out, as previously described [42,43].

## Results

Quantitative PCR on four of the five samples with sufficient DNA extract volume indicate a 14,000 to 300,000-fold difference in the amount of recovered nuclear DNA:mtDNA, highlighting the greater potential for mtDNA typing in degraded remains. The total nuclear DNA in all four samples was very low (<2 pg/μL). Subsequently, all four samples only produced partial STR profiles using low copy number techniques (34 cycles of PCR and reduced reaction volumes with higher concentrations of *Taq* DNA polymerase) (Table 2). Locus dropout was observed in each degraded sample analysed for nuclear STR typing. Only the positive control DNA produced a full STR-DNA profile, which matched the reference profile at all ten loci examined (Table 2). All negative controls were blank.

**Table 2:** Mitochondrial and nuclear quantitative PCR (qPCR) and STR typing result

**Sample ID**	**Mitochondrial DNA**	**Nuclear DNA**				
	**qPCR**	**qPCR**	**qPCR**	**STR typing (Profiler Plus kit, 10 loci) 34 cycles**		
	**77 bp target (copies/μL)**	**67 bp target (copies/μL)**	**67 bp target (ng/μL)**			
				**Number of loci**	**Percentage genotyping success**	**None, partial or full profile obtained**
4464B	15,727	<1 copy (0.54)	0.001794	8	80%	Partial
10730A	62,592	0	0	Amel only	10%	Partial
8727C	350	<1 copy (0.025)	0.000082	3	30%	Partial
11995A	2,715	<1 copy (0.0091)	0.00003	2	20%	Partial
9210A	n/a	n/a	n/a	n/a	n/a	n/a
Negative control (H20)	0	<1 copy (0.034)	0.000111	0	0%	None
Female positive control	7,896,895	543	1.79213	10	100%	Full

n/a, non-applicable (The sample was excluded from testing due to the low quantity of DNA extract. Quantitative PCR data is presented as a geometric average using triplicate values.).

**Figure 3:**
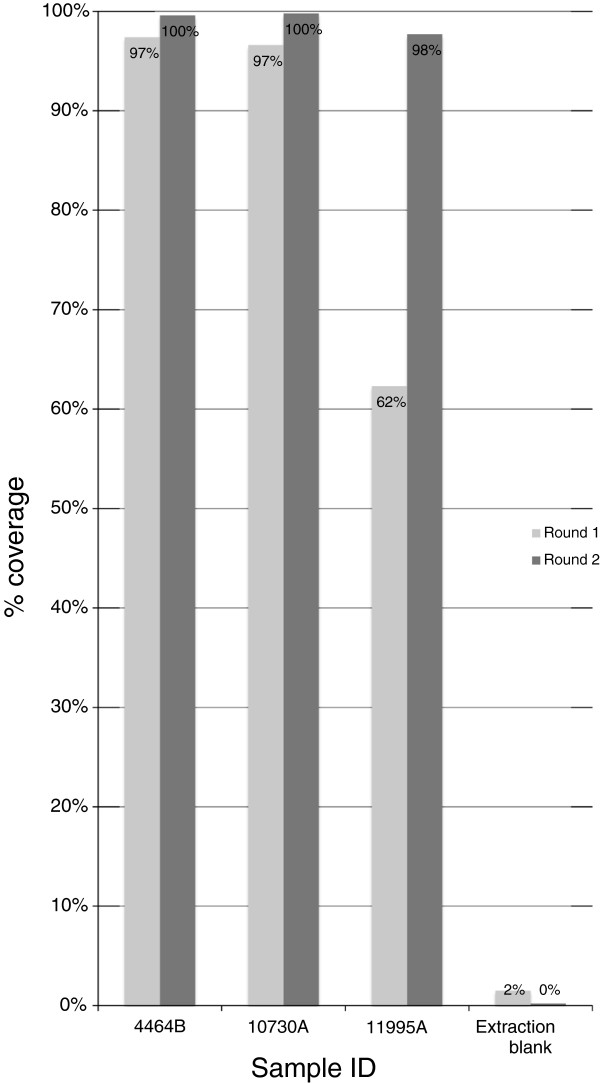
Percent coverage of human mitochondrial genomes sequenced after one (light grey bars) and two (dark grey bars) rounds of enrichment for three degraded samples and an extraction blank.

In contrast, a higher concentration of mitochondrial DNA was detected in all four samples using qPCR (Table 2). DNA library preparation, mtDNA enrichment and NGS were completed for all five samples. After one round of hybridisation and enrichment we obtained 96% to 97% of the mitochondrial genome at an average 15 to 18-fold coverage from two well-preserved samples but only 62% of the mitochondrial genome at an average 1-fold coverage on a poorly preserved sample (Table 3, Figure 3). However, after two rounds of hybridisation and enrichment we obtained 98% to 100% of the mitochondrial genome at an average 1646-fold coverage from all five samples, irrespective of morphological preservation of the sample (Table 3, Figure 3). Complete or near complete mitogenomes were recovered from samples with as few as 350 copies/μL of the 77 bp mtDNA fragment.

**Table 3:** Ion Torrent PGM whole mitochondrial DNA sequencing data, after one and two rounds of enrichment

**ACAD ID**	**Rounds capture**	**Percentage coverage**	**Average length (± SD)**	**Number of bases covered**	**Unique reads**	**Mean X coverage (± SD)**	**Haplotype**
4464B	1	97.4%	42 (± 11.0)	16,136	7,069	18 (± 12.3)	HV0e
4464B (ancient)	2	99.60%	45 (± 11.7)	16,507	14,837	40 (± 19.8)	HV0e
10730A	1	96.6%	43 (± 11.3)	16,004	5,760	15 (± 10.7)	B2b
10730A (ancient)	2	99.80%	47 (± 12.2)	16,540	16,138	46 (± 20.2)	B2b
11995A	1	62.3%	40 (± 10.2)	10,321	553	1 (± 1.6)	Low coverage
11995A (forensic)	2	97.70%	45 (± 12.9)	16,188	6,012	16 (± 11.2)	H1a
Extraction blank	1	1.5%	41 (± 18.4)	248	6	0	n/a
Extraction blank (EBC11049)	2	0.20%	0	28	1	0	n/a
8727C (forensic)	2	99.50%	43 (± 9.3)	16,487	7,756	20 (± 9.7)	U5a2a1fNEW
9210A (forensic)	2	100%	65 (± 20.4)	16,569	7,865	31 (± 14.1)	J1c12

ACAD, Australian Centre for Ancient DNA; n/a, non-applicable.

**Figure 4:**
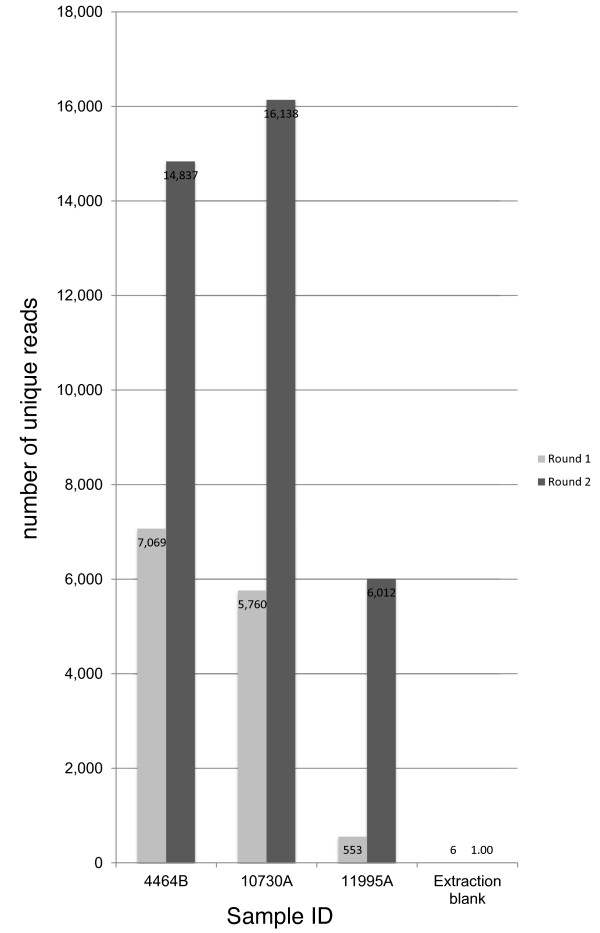
Number of unique sequencing reads mapped to the Reconstructed Sapiens Reference Sequence mitochondrial genome after one (light grey bars) and two (dark grey bars) rounds of enrichment for three degraded samples and an extraction blank.

Two rounds of enrichment substantially improved the number of unique reads that mapped to the mtDNA genome (from 2- to 11-fold) (Figure 4, Additional file 1: Figure S1) and the average redundancy per site of the genome (from 1 to 18× to 16 to 46×) but did not alter the mean fragment length of mtDNA recovered (42 bp after one round and 45 bp after two rounds) (Table 3). The second round of enrichment proved to be particularly important for the less well-preserved sample 11995A, for which it provided an 11-fold increase in the total number of unique reads, which also substantially improved the coverage from 62% to a near complete mitochondrial genome (98%) and therefore allowed an unambiguous haplotype designation (Table 3).

Coverage was uneven across all five samples after one and two rounds of enrichment (Additional file 1: Figure S1). This variation in coverage has been reported previously for modern and ancient human and Neanderthal mtDNA genomes [5,24,44-46] and is positively correlated with GC content. This may be due to loss (denaturation) of short AT-rich sequences before or during the library preparation [5,46].

Damage patterns in all samples followed expectations for degraded DNA, with a larger than usual amount of deaminated cytosine residues accumulated towards the ends of the molecules. In addition, we could observe a high frequency of indels, a well-identified homopolymer sequencing error characteristic of PGM technology [47-50]. However, indels were randomly distributed and did not affect the final consensus sequences, as each called position was covered with enough depth to prevent false-positive base calls.

**Figure 5:**
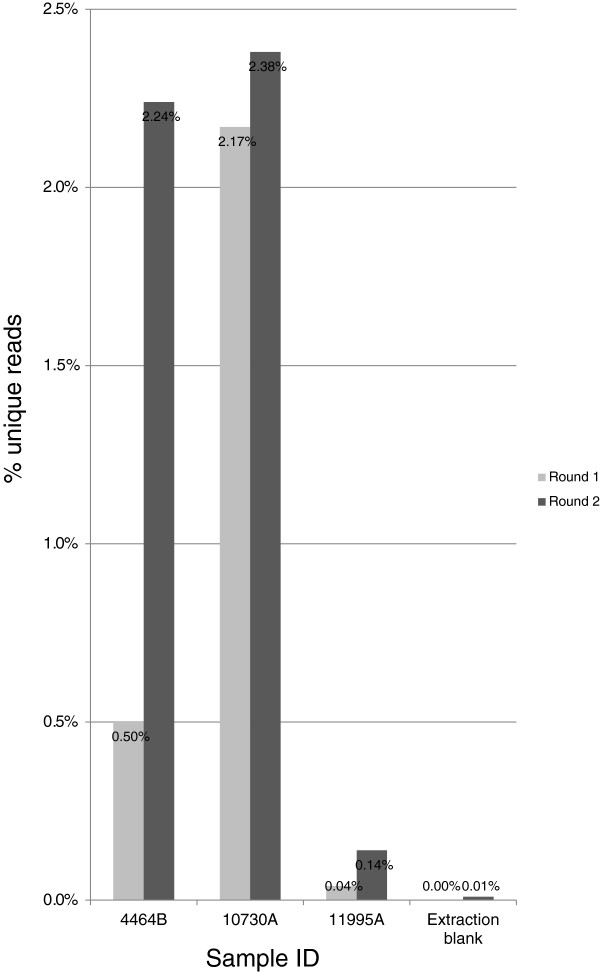
Percentage of unique sequencing reads that map to the Reconstructed Sapiens Reference Sequence mitochondrial genome after one (light grey bars) and two (dark grey bars) rounds of enrichment for three degraded samples and an extraction blank.

Stringent quality filtering during analysis removed a large proportion of the total reads for each sample. Post-filtering provided on average, across all samples, a very small proportion of unique mapped reads vs total reads (0.04% to 2.4%) (Figure 5). However, the pattern of mapped reads had an adequate level of coverage for each sample to allow detection of variants in the mitochondrial genome. Traditional HVS I sequencing and coding region SNP mini-sequencing of all five samples produced identical results to those obtained by whole mtDNA genome sequencing.

## Discussion

Low amounts of DNA combined with high levels of damage and fragmentation make STR typing of degraded samples challenging. DNA capture coupled with next-generation sequencing can retrieve whole mitochondrial genome sequences from degraded samples when nuclear DNA is below detection levels. Despite high levels of DNA decay in skeletal remains, whole mtDNA genome sequencing is possible due to the copy-number advantage and reduced rate of fragmentation of mtDNA (compared with nuclear DNA) combined with the ability to capture and sequence DNA fragments in the 20 bp to 70 bp range. Quantitative PCR can be used to determine the amount of DNA available from extracted materials and will indicate the likelihood of obtaining a nuclear STR-DNA profile from a degraded sample. This is of particular importance in cases where total nuclear DNA quantity is <100 pg, which reduces the likelihood of obtaining a full nuclear STR DNA profile even when applying LCN techniques. In contrast, near complete mitochondrial genome sequences can be obtained with a single round of enrichment from samples with >10,000 77 bp mtDNA copies/μL and with two rounds of enrichment from samples with <3,000 77 bp mtDNA copies/μL. Our work builds on previous in-solution capture-based enrichment methodologies [21,24-26] and demonstrates the importance of using multiple rounds of enrichment to improve mtDNA genome recovery from samples with low amounts of endogenous DNA. Repeating the enrichment process on samples with very low amounts of mtDNA can more than double the number of unique reads and average coverage, and substantially improve the overall coverage of the mtDNA genome (Additional file 1: Figure S1). The methodology has the ability to capture DNA templates that are damaged and fragmented (<100 bp in length) (Figure 2) and that are generally difficult to recover using traditional methods of PCR-based amplification and sequencing [51]. This is of particular importance in cases where DNA has been exposed to prolonged heat, moisture, ultraviolet light and microbial attack, which generally results in template fragmentation (in extreme cases there can be no surviving endogenous DNA templates >100 bp) [52].

Two common concerns with mitochondrial DNA testing can be eliminated or reduced using this whole mtDNA genome sequencing approach. Traditional HVS I/II sequencing requires 2 to 12 separate PCR amplifications and up to 24 separate DNA sequencing reactions. This multi-tube, multi-step approach introduces the potential for sample mix-up during laboratory processing and increases the risk of introducing contaminating DNA. Our whole mtDNA genome approach eliminates this risk, massively reducing opportunities for sample mix-up, while the barcoded adapters ligated to the DNA provide an additional means to eliminate (or identify and screen out) contamination introduced in later steps. In addition, barcoding allows many samples to be pooled for high-throughput screening efforts and can reduce the cost of sequencing.

DNA capture and related approaches have been shown to give preferential enrichment of short endogenous DNA templates over longer exogenous contaminant DNA in a sample [7]. This is particularly important where small quantities of endogenous DNA in a sample have become saturated by larger quantities of exogenous contamination (human and microbial), consequently leading to poor PCR amplification, mistyping of target loci via artefacts or even complete PCR amplification failure [1].

Traditional forensic and archaeogenetic studies using mtDNA have relied on HVS I/II sequencing. However, this relatively short sequence has limited resolving power and can fail to discriminate between distinct maternal lineages [20]. Outside the control region, coding region SNPs provide additional resolution and discriminatory power [20,53]. To date, this additional information has been obtained via case [20,54], region [55,56], continental [57] or haplogroup [58] specific SNP multiplex assays. In contrast, our whole mtDNA genome sequencing approach is a universal solution for obtaining high-resolution mtDNA data, which can discriminate between closely related maternal lineages. However, although our methodology provides a mechanism to generate whole mtDNA genome sequences from difficult and degraded samples, there is a clear need for the parallel development of high-quality mitochondrial genome databases [20,53].

Complete mitochondrial genomes sequences can aid human identification efforts by placing an individual into specific haplotypes based on private SNPs. This high-resolution discrimination can be used to include or exclude closely related maternal lineages [21], especially in populations with high frequencies of particular haplotypes. By resorting to whole mtDNA sequencing, we were able to gain additional haplogroup and haplotype resolution relative to traditional HVS I/II sequencing. This information has already proved critical in a comparison with maternal relatives in a case where the HVS I/II sequence alone could not exclude a maternal relationship. Our approach could assist large-scale identification efforts when more comprehensive mtDNA reference databases become available to the forensic community.

Validation studies have confirmed that mtDNA typing is a reliable means of forensic identification [59]. However, a worldwidewide effort will be required with labs collaborating and producing large databases, estimating the frequency of particular mtDNA haplotypes and improving the statistical basis of the databases. In the meantime, techniques used to sequence whole mtDNA in archaeological and population studies will continue to advance at a rapid pace.

## Conclusions

In-solution capture-based whole mitochondrial genome sequencing immortalises the limited and important contents of the DNA extract in the form of a DNA library, and is followed by targeted enrichment of mtDNA sequences. The application of these methods using hybridisation enrichment and NGS has led to the reliable genotyping of human remains for which standard nuclear PCR protocols had been unsuccessful. This result indicates that the technique can be applied to obtain whole mitochondrial genomes even from particularly challenging samples. Additionally, as NGS platforms become more affordable and widely available and with the advent of DNA library, barcoding (to monitor contamination and allow multiple samples to be processed), new methods for mtDNA analysis should be considered.

## Abbreviations

ACAD: Australian Centre for Ancient DNA; bp: base pair; HVS: Hypervariable region; ISP: Ion sphere particle; kb: kilobase; LCN: Low copy number; mtDNA: mitochondrial DNA; NGS: Next-generation sequencing; PCR: Polymerase chain reaction; PGM: Personal Genome Machine; qPCR: quantitative PCR; RFU: Relative fluorescence units; SSC: Saline sodium citrate; SDS: Sodium dodecyl sulphate; SNP: Single nucleotide polymorphism; STR: Short tandem repeat.

## Competing interests

The authors declare that they have no competing interests.

## Authors’ contributions

JELT co-developed the protocol, processed samples, performed next-generation sequencing, co-analysed data and wrote the manuscript. PB designed and developed the DNA extraction, library preparation and targeted enrichment protocol, co-developed the underlying research concept and assisted manuscript preparation. BL processed sample extraction and co-developed the analytical pipeline for data analysis with JS. BL and JS performed data analysis and assisted with manuscript preparation. WH contributed to the experimental design, provided archaeological samples, processed sample extractions and library preparations and assisted with manuscript preparation. AC co-developed the underlying concept, contributed to the experimental design and assisted with manuscript preparation. JA provided forensic samples, processed sample extractions, contributed to the experimental design and assisted with manuscript preparation. All authors read and approved the final manuscript.

## Supplementary Material

Additional file 1 **Detailed description of the methods used to extract DNA from bone samples, prepare capture-bait library and prepare libraries from degraded DNA.** Primer sequences are shown in Tables S2, S3, S4, S5, and S6. Mitochondrial genome haplotypes for laboratory staff and degraded bone samples are shown in Tables S1 and S8, respectively. Details of Ion Torrent sample barcoding and sequencing runs are shown in Table S7. Mapping of individual sequence reads to the reference mitochondrial genome for all five degraded samples are shown in Figure S1. Click here for file
