# Supplementary material for: DNA capture and next-generation sequencing can recover whole mitochondrial genomes from highly degraded samples for human identification
Source: Investig Genet. 2013 Dec 2;4:26. doi: 10.1186/2041-2223-4-26 (PMC3879034; doi:10.1186/2041-2223-4-26)
Supplement: Additional file 1 — Detailed description of the methods used to extract DNA from bone samples, prepare capture-bait library and prepare libraries from degraded DNA. Primer sequences are shown in Tables S2, S3, S4, S5, and S6. Mitochondrial genome haplotypes for laboratory staff and degraded bone samples are shown in Tables S1 and S8, respectively. Details of Ion Torrent sample barcoding and sequencing runs are shown in Table S7. Mapping of individual sequence reads to the reference mitochondrial genome for all five degraded samples are shown in Figure S1. [file 2041-2223-4-26-S1.docx]

**Supplementary information (SI)**

Description of technique

The method of enriching target DNA prior to sequencing utilises a DNA sequencing library (extracted DNA fragments with universal adaptors ligated to the ends of the molecules) as the template for enrichment. With the aim to obtain sequences from the whole mitochondrial genome of poor quality samples hybridisation was used to capture and enrich small fragments (<100 base pairs) of endogenous DNA, containing adaptor sequences. For the purpose of enriching human DNA from mixtures containing non-human DNA, present day human mitochondrial DNA probes were used to hybridise to endogenous targets of interest. A biotin tag was added to the 3’ end of the probe DNA with the aim of ‘pulling-out’ the complementary sequences of human library DNA, while the probe DNA-library complex was fixed to streptavidin-coated paramagnetic beads and subjected to several subsequent stringency washes. The enriched library DNA was subsequently eluted from the stable probe fixed to magnetic beads using a strand displacing enzyme at optimum temperature. The targeted enrichment of complex adaptor-ligated DNA libraries can be performed with multiple samples in parallel, when a sample specific barcode is added to the ends of the universal adaptor sequences, if so required for high-throughput sequencing.

**Materials and methods (SI)**

**DNA extraction**

Preparation of bone samples for DNA extraction was carried out as previously described [1]. Silica-based extraction, previously developed and optimized in-house, was used to recover small DNA fragments <100bp [1].

1. 200mg of bone powder was incubated over-night at 37C in a rotating oven consisting of 4.44mL lysis buffer (0.5M EDTA pH 8.0; 0.25mg/uL proteinase K; and 0.5% N-lauroylsarcosine).
2. Day two, the lysed material was centrifuged at 4,600 rpm (1min) and supernatant was transferred to a new 50mL falcon. Binding of DNA to silica involved an overnight incubation, in a rotating oven at room temperature in 125uL medium-sized silica suspension and 16mL of in-house binding buffer (13.5mL QG buffer (Qiagen), 1X Triton, 20mM NaCl, 0.2M acetic acid (all Sigma-Aldrich)). The pH-indicator in solution monitored a neutral environment at which DNA binds to silica.
3. Day three, the sample was centrifuged for 1min at 13,000rpm to pellet silica particles and the supernatant was discarded. Pellet was transferred to a 1.5mL tube and subjected to three washes in 1mL 80% ethanol, centrifuged for 1min at 13,000rpm and supernatant discarded. The pellet was air dried (30min), resuspended in 200uL (pre-warmed to 50°C) TE buffer (10mM Tris, 1mM EDTA) and incubated for 10min. After pelleting (1min at 13,000rpm) the supernatant was collected, aliquoted and stored at -18°C until further use.

**Preparation of probe bait (in a separate facility to library DNA preparation)**

1. Probe DNA was generated from a present-day individual (of a known haplotype). Total genomic DNA was extracted from a buccal swab using the DNeasy Blood & Tissue Extraction kit (Qiagen), following manufacturer’s instructions. The whole mitochondrial genome was amplified in eight separate 25uL LR-PCR reactions (Roche, Long Range Expand kit) using PCR primers (Table S2). Two overlapping 8Kb fragments were generated spanning 16,569bp. In total 42 cycles of PCR were carried out using the Bio-Rad Tetrad Thermal Cycler. Cycling conditions included: initial denaturation at 92° for 2 mins; 10 cycles of 92°C for 10sec, 60°C for 15sec, 68°C for 8min 40sec; followed by 32 cycles of 92°C for 10sec, 60°C for 15sec, 68°C for 8min 40sec – increasing by 15sec/cycle; final extension at 68° for 10mins; followed by a hold at 4°C.
2. PCR reactions were pooled for each product and loaded on a 1% agarose gels. Bands were visualised by ethidium bromide staining prior to excision of the correct-sized band under UV light with a clean, sharp scalpel blade. Purification of bands was carried out using the QIAquick Gel Extraction Kit (Qiagen) and eluted in 30uL as per the manufacturer’s instructions.
3. Quantification was performed using a Nanodrop 2000 (Thermo Scientific).
4. DNA was diluted to 120uL total volume with distilled water as a requirement for the sonicator prior to shearing. Fragmentation of DNA to the desired size range was achieved by sonication (Microtip sonicator, Thomas Optical & Co. Pty. Ltd) at high speed, amplitude 6, for 4min, no ice. Post sonication, DNA was purified and concentrated using the QIAquick PCR Purification Kit (Qiagen) and eluted in 30uL as per the manufacturer’s instructions.
5. The resulting DNA was analysed on a 1% agarose gel, with the resulting smear in the size range 200-600bp. DNA concentration was estimated using gel electrophoresis against quantified size markers (HyperLadder™ V, Bioline) and a Nanodrop 2000 (Thermo Scientific). DNA from both halves of the mitochondrial genome were pooled to produce an equimolar concentration of probe DNA across the genome, prior to biotinylation with Biotin-16-ddUTP and the enzyme Terminal Transferase (TdT) (NEB).
6. Prior to 3’ end-labelling, sonicated probe DNA was made single stranded by heating at 95°C for 5min then immediately placed on ice for 5min. Reactions for the 3’ end-labelling of probe DNA were performed at 50uL final volumes comprising: 50mM Potassium Acetate; 20mM Tris-Acetate pH 7.9; 10mM Magnesium Acetate; 0.25mM Cobalt Chloride; sonicated mitochondrial probe DNA at 10pmol of 3’ ssDNA ends; 0.1mM Biotin-16-ddUTP (Enzo); and 40U Terminal Transferase enzyme (New England Biolabs). The thermocycling profile was: 37°C for 60min; then 70°C for 10min.
7. DNA was purified using the Nucleotide Removal Kit spin columns (Qiagen) and eluted into 30uL as per the manufacturer’s instructions. The rationale was that 3’-Biotin-16-ddUTP labelling the probe would prevent probe molecules from extending under any circumstances.

**Library Preparation**

1. Preparing adaptor mix. Hybridising Uni-Hyb Af and Uni-Hyb Ar (Table S3) at 95C for 2min, 75°C for 20sec, followed by a ramp from 75°C to 10°C at 2°C/min increments. Hybridising Uni-Hyb Bf and Uni-Hyb Bf (Table S3) at 95C for 2min, then 75°C for 20sec, followed by a ramp from 75°C to 10°C at 2°C/min increments.
2. DNA end repair and phosphorylation. Between 5-25μL of DNA extract was added to 100uL final volume comprising 1x T4 DNA Ligase Buffer, 250μg/mL rabbit serum albumin (RSA; Sigma), 400μM of each dNTP (Invitrogen), 50U T4 Polynucleotide Kinase (New England Biolabs, NEB), 10U DNA Polymerase I, Large (Klenow) Fragment (NEB), and 15U T4 DNA Polymerase (NEB). Thermocycling profiles consisted of 1 cycle of 25°C for 15 min, 37°C for 15min, and 12°C for 15min. At 12°C, 10μL of 0.5M EDTA pH8.0 (Sigma) was added, followed by 550uL Qiagen Buffer PB1.
3. DNA purification. Mini-elute spin column purification (Qiagen) was performed as per the manufacturer’s instructions. Adaptor ligation reactions were performed at 60μL final volume with final reactions comprising of 0.8 x Quick ligation buffer (NEB), 2μM Adaptor UniHyb-A, 2μM Adaptor UniHyb-B, and 4,000U T4 DNA Ligase (NEB). The thermocycling profiles consisted of 20 cycles of 24°C for 1min, 16°C for 30sec, and 8°C for 30sec. 300μL Qiagen Buffer PB1 was added to each reaction. DNA was isolated from the rest of the reaction components using Mini-elute spin columns (Qiagen) as per the manufacturer’s instructions.
4. Polymerase ‘fill-in’ reactions. To create fully double-stranded adaptor-tagged DNA, reactions at 30μL final volume comprised of: 1 x Thermopol Buffer (NEB), 250μM of each dNTP, 16U Bst DNA Polymerase, Large Fragment (NEB) and purified library DNA. The thermocycling profile was 37°C for 30min. 150μL Qiagen Buffer PB1 was subsequently added to each reaction. DNA was purified using Mini-elute spin columns (Qiagen) and eluted into 21μL as per the manufacturer’s instructions.
5. PCR amplification. 3 x 44μL reaction volumes per original sample/extract were amplified (to reduce amplification bias) with 7μL of eluted DNA added per tube. Final reactions conditions comprised of 1x AmpliTaq Gold buffer II, 2.5mM MgCl2, 2.5U AmpliTaq Gold (Applied Biosystems), 250μM of each dNTP (Invitrogen), and 0.5μM of each PCR primer (Table S4). The thermocycling profile consisted of 94 °C for 11 min, followed by 12 cycles of 30sec at 95°C, 30sec at 60°C and 1min (+2 sec/cycle) at 72°C, followed by a final 10min at 72°C. The 3 x 44μL volume reactions were pooled. From each original sample/extract DNA library pool, 2.5μL was then added to 4 x 35μL reactions comprising: 1X AmpliTaq Gold buffer II, 2.5mM MgCl2, 250μM of each dNTP, 1.0U AmpliTaq Gold (Applied Biosystems); and 0.5μM of each PCR primer (Table S4).
6. Re-amplification purification. 4 x 35uL amplification reactions were purified using MinElute spin columns (Qiagen) and eluted in 15μL as per the manufacturer’s instructions. The amplification products were sized and quantified via gel electrophoresis against quantified size markers (HyperLadder™ V, Bioline) and a Nanodrop 2000 (Thermo Scientific).

Table S1. MtDNA haplotypes and SNPs of staff compared to the Revised Sapiens Reference Sequence.

| Staff ID | | Haplotype | | polymorphic sites |
| --- | --- | --- | --- | --- |
| ADL1 | | H3 | | G73A, C146T, 195T, A247G, (315.1C), (523.AC), A769G, A825t, A1018G, G2706A, A2758G, C2885T, T3594C, G4104A, T4312C, T6776C, T7028C, G7146A, T7256C, A7521G, T8468C, T8655C, G8701A, C9540T, G10398A, T10664C, A10688G, C10810T, C10873T, C10915T, A11590G, A11719G, A11914G, T12705C, G13105A, G13276A, T13506C, T13650C, A14687G, T14766C, T15908C, A16129G, T16187C, C16189T, T16223C, G16230A, T16278C, C16311T, T16325C |
| ADL3 | | H1a4 | | C146T, C152T, C195T, A247G, (309.1C, 315.1C), (523.AC), A769G, A825t, A1018G, G2706A, A2758G, C2885T, G3010A, T3594C, G4104A, T4312C, T7028C, G7146A, T7256C, A7521G, T8468C, T8655C, G8701A, A9341t, C9540T, G10398A, T10664C, A10688G, C10810T, C10873T, C10915T, A11719G, A11914G, T12705C, G13105A, G13276A, T13506C, T13650C, T14766C, A16129G, A16162G, T16187C, C16189T, T16223C, G16230A, T16278C, C16311T |
| ADL4 | | H1be | | G73A, C146T, C152T, C195T, A247G, (309.2C, 315.1C), (523.AC), A769G, A825t, A1018G, G2706A, A2758G, C2885T, G3010A, T3594C, G4104A, T4312C, T7028C, G7146A, T7256C, A7521G, T8468C, T8655C, G8701A, A9341t, C9540T, G10398A, T10664C, A10688G, A10750G, C10810T, C10873T, C10915T, A11719G, A11914G, T12705C, C13035T, G13105A, G13276A, T13506C, T13650C, T14766C, A16129G, T16187C, C16189T, C16192T, T16223C, G16230A, T16278C, C16311T |
| ADL6 | | H1z1 | | G73A, C146T, C152T, C195T, A247G, (309.2C, 315.1C), C327T, (523.AC), A769G, A825t, A1018G, G2706A, A2758G, C2885T, G3010A, T3594C, G4104A, T4312C, T7028C, G7146A, T7256C, A7521G, T8468C, T8655C, G8701A, C9540T, G10398A, T10632C, T10664C, A10688G, C10810T, C10873T, C10915T, C11428T, A11914G, T12705C, G13105A, G13276A, T13506C, T13650C, T14766C, A16129G, T16187C, T16223C, G16230A, T16278C |
| ADL7 | | H1 | | G73A, C146T, C195T, A247G, (309.1C, 315.1C), (523.AC), A769G, A825t, A1018G, G2706A, A2758G, C2885T, G3010A, T3594C, G4104A, T4312C, T7028C, G7146A, T7256C, A7521G, T8468C, T8655C, G8701A, C9540T, G10398A, T10664C, A10688G, C10810T, C10873T, C10915T, A11719G, A11914G, T12705C, G13105A, G13276A, T13506C, T13650C, T14766C, A16129G, T16187C, C16189T, T16223C, G16230A, T16278C, C16311T |
| Probe DNA | | J1c8a | | C146T, C152T, G185A, C195T, G228A, A247G, C295T, C462T, T489C, (523.AC), A769G, A825t, A1018G, A2758G, C2885T, G3010A, T3594C, G4104A, T4216C, T4312C, G7146A, T7256C, A7521G, T8468C, T8655C, G8701A, A9052G, C9540T, T10084C, G10398A, T10664C, A10688G, C10810T, C10873T, C10915T, A11251G, A11914G, A12612G, T12705C, G13105A, G13276A, T13506C, T13650C, G13708A, T14798C, C15452a, C16069T, T16126C, A16129G, T16187C, C16189T, T16223C, G16230A, C16261T, A16265G, T16278C, C16311T, G16319A, C16519T |
|  | |  |  |  |
|  | |  |  |  |

Table S2. Long Range PCR primers

| Primer | Sequence 5'-3' |
| --- | --- |
| L06363 | ACCATCTTCTCCTTACACCTAGCAG |
| H14799 | GGTGGGGAGGTCGATGA |
| L14759 | AGAACACCAATGACCCCAATAC |
| H06378 | GATGAAATTGATGGCCCCTAA |

Table S3. Universal hybridisation adapters used to make hybridisation primers

| UniHyb Adapters | Sequences 5'-3' |
| --- | --- |
| UniHyb Adapter Af | GGTGTTGTTAGGAATGCGAGA |
| UniHyb Adapter Ar | TCTCGCATTCCTAA |
| UniHyb Adapter Bf | AGGATAGGTCGTTGCTGTGTA |
| UniHyb Adapter Br | TACACAGCAACGA |

Table S4. Universal hybridisation primers

| Primers | Sequences 5'-3' |
| --- | --- |
| UniHyb Primer A | GGTGTTGTTAGGAATGCGAGA |
| UniHyb Primer B | AGGATAGGTCGTTGCTGTGTA |

Table S5. Quantitative PCR (qPCR) primers

| Primers | Target length and fragment | Sequences 5'-3' |
| --- | --- | --- |
| L13258 | MtDNA  77 bp target | ATCGTAGCCTTCTCCACTTCAA |
| H13295 |  | AGGAATGCTAGGTGTGGTTGGT |
| HomoSap_CSF_STR_F | Nuclear DNA  67bp target | gggcagtgttccaacctgag |
| HomoSap_CSF_STR_R |  | GAAAACTGAGACACAGGGTGGTTA |

Table S6. Ion Torrent PCR primers

| Primers | Sequences 5'-3' |
| --- | --- |
| LlyfrITOB | CCTCTCTATGGGCAGTCGGTGATAGGATAGGTCGTTGCTGTGTA |
| LlyfrITOA1 | CCATCTCATCCCTGCGTGTCTCCGACTCAGAAAAAGGTGTTGTTAGGAATGCGAGA |
| LlyfrITOA2 | CCATCTCATCCCTGCGTGTCTCCGACTCAGTAATTGGTGTTGTTAGGAATGCGAGA |
| LlyfrITOA3 | CCATCTCATCCCTGCGTGTCTCCGACTCAGTATATGGTGTTGTTAGGAATGCGAGA |
| LlyfrITOA4 | CCATCTCATCCCTGCGTGTCTCCGACTCAGAATTAGGTGTTGTTAGGAATGCGAGA |

Table S7. Ion Torrent barcoded primers and chip details

| ACAD sample ID | Ion Torrent barcoded primers | Sequencing run details |
| --- | --- | --- |
| 8727 | ITOA1 + ITOB | run individually on 316 chip |
| 11995 | ITOA3 + ITOB | pooled prior to run on 316 chip |
| 9210 | ITOA1 + ITOB | run individually on 316 chip |
| 4464 | ITOA1 + ITOB | pooled prior to run on 316 chip |
| 10730 | ITOA2 + ITOB | pooled prior to run on 316 chip |
| EBC | ITOA4 + ITOB | pooled prior to run on 316 chip |

Table S8. MtDNA haplotypes and SNPs of samples compared to the Revised Sapiens Reference Sequence.

| ACAD ID | Haplotype | SNPs | Private SNPs |
| --- | --- | --- | --- |
| 4464B | HV0e ? | **72C**,**73A,146T,152T,182C!**,**195C!,247G**,523A,524C,**769G,825T, 1018G,2758G,2885T,3594C,4104A,4312C,7146A,7256C,7521G,8468C,8655C**,**8701A,9540T,10398A**,**10609C,10664C,10688G,10810T**,**10873T,10915T**,**11719G,11914G**,**12705C,13105A,13276G,13506C,13650C**,**14766C,15301G!**,**15454C,16129G,16187C,16189T**,**16223C,16230A,16278C**,**16298C,16311T,16519T** | One diagnostic SNP 16311C! missing for HV0e |
| 10730A | B2b | 131C,**146T**,**152T,182C!**,**195T**,**247G**,**499A**,523A,524C,**769G,825T,827G**,**1018G,2758G,2885T**,**3547G,3594C**,4013T**,4104A**,**4312C**,**4820A**,**4977C,6473T**,**6755A**,**7146A,7256C,7521G,8281-8289d,8468C,8655C,8701A,9540T**,**9950C,10398A,10664C,10688G,10810T,10873T,10915T,11177T,11914G,12705C**,**13105A,13276A,13506C**,**13590A,13650C**,13708A,14634C,**15301G!,15535T**,15784C,**16129G**,16183C,**16187C,16189C!**,**16217C**,**16223C,16230A,16278C,16311T** | 131C,13708A,14634C,15784C,16183C |
| 11995 | H1a | **73G!,146T**,152C!,**182C!,195T,247G**,523A,524C,**769G,825T,1018G**,**2706A,2758G**,**2885T,3010A,3594C,4104A,4312C**,4541A,**7028C,7146A**,**7256C,8468C,8655C**,**8701A,9540T,10398A,10664C,10688G,10810T,10873T,10915T**,**11719G**,**11914G,13105A,13276A,13506C,13650C**,**14766C**,14769G,**15301G!,16129G,16162G,16187C,16189T**,16223C**,16230A,16278C,16311T** | 152C!,4541A,14769G |
| 8727 | U5a2a1f NEW | **146T,152C!,182C!**,**195T**,**247G**,523A,524C,**769G**,**825T**,**1018G**,**2758G**,**2885T**,**3197C**,**3594C**,**4104A**,**4312C**,**7146A**,**7256C,7521G,8468C,8655C**,**8701A**,9180G,**9477A**,**9540T,10398A**,**10664C**,**10688G**,**10810T**,**10873T**,**10915T**,**11467G**,**11914G,12308G**,**12372A**,**12705C**,**13105A**,**13276A**,**13506C**,**13617C**,**13650C**,**13827G**,**13928C,14793G**,**15301G!,16114A**,**16129G**,**16187C**,**16189T**,**16192T**,**16223C**,**16230A**,**16256T,16270T**,**16278C**,**16294T,16311T**,16519T**,16526A** | 9180G,16519T |
| 9210A | J1c12 | **146T,152T,182C!**,**185A**,**189G,195T**,217C,**228A**,**247G**,**295T**,**462T,489C**,523A,524C,**769G**,**789C**,**825T,1018G,2758G,2885T**,**3010A**,3592A,**3594C**,4084A,**4104A**,**4216C,4312C,7146A,7256C,7521G,8468C,8655C**,**8701A,9540T**,**10398G!**,**10664C,10688G,10810T**,**10873T,10915T**,**11251G**,**11914G**,**12612G**,**12705C**,**13105A,13276A,13506C,13650C,13708A**,**14798C**,**15301G!**,**15452A**,**16069T**,**16126C**,**16129G,16187C,16189T**,**16223C,16230A**,**16261T**,**16278C,16311T**,16519T | 217C,3592A,16519T |

Figure S1. Short DNA sequences obtained after one and/or two rounds of capture-based enrichment of degraded DNA from five forensic and ancient samples mapped against the Reconstructed Sapiens Reference Sequence. Each black bar represents a single unique read (mean length 40-65 bp). The vertical scale bar shows the number of unique reads covering each region of the mtDNA genome.

Figure S1.(cont.)

**Reference**

1. Brotherton P, Haak W, Templeton J, Brandt G, Soubrier J, Adler CJ, Richards SM, Der Sarkissian C, Ganslmeier R, Friederich S, Dresely V, van Oven M, Kenyon R, Van der Hoek MB, Korlach J, Luong K, Ho SYW, Quintana-Murci L, Behar D, Meller H, Alt KW, Cooper A and the Genographic Consortium: **Neolithic mitochondrial haplogroup H genomes and the genetic origins of Europeans.** *Nat Commun* 2013, **4:**1764.
